# Incidence and outcomes of chronic total occlusion percutaneous coronary intervention in the Netherlands: data from a nationwide registry

**DOI:** 10.1007/s12471-020-01521-y

**Published:** 2020-12-02

**Authors:** A. van Veelen, B. E. P. M. Claessen, S. Houterman, L. P. C. Hoebers, J. Elias, J. P. S. Henriques, P. Knaapen, the PCI Registration Committee of the Netherlands Heart Registration

**Affiliations:** 1 Department of Cardiology, Heart Centre, Amsterdam Cardiovascular Sciences, Amsterdam UMC, University of Amsterdam, Amsterdam, The Netherlands; 2 Department of Cardiology, Northwest Clinics, Alkmaar, The Netherlands; 3 Netherlands Heart Registration, Utrecht, The Netherlands; 4 Department of Cardiology, Heart Centre, Amsterdam Cardiovascular Sciences, Amsterdam UMC, VU University, Amsterdam, The Netherlands

**Keywords:** Chronic total occlusion, Percutaneous coronary intervention, Mortality, Renal insufficiency

## Abstract

**Background:**

Patients with chronic total coronary occlusions (CTO) are at increased risk for poor clinical outcomes. We aimed to determine the incidence of CTO percutaneous coronary intervention (PCI) and to identify CTO patients at risk for cardiac events in the nationwide Netherlands Heart Registration (NHR).

**Methods:**

We included all PCI procedures with ≥1 CTO registered in the NHR from January 2015 to December 2018, excluding acute interventions. We used multivariable logistic regression of baseline characteristics to calculate the risk for events as odds ratios (OR) with 95% confidence intervals (CI).

**Results:**

Of the PCIs performed during the study period, 6.3% (8,343/133,042) were for CTOs, with the percentage increasing significantly over time from 5.9% in 2015 to 6.6% in 2018 (*p* < 0.001). Coronary artery bypass grafting <24 h was carried out in 0.3%, and the only significant predictor was diabetes mellitus (OR 2.97, 95% CI 1.04–8.49, *p* = 0.042). Myocardial infarction (MI) <30 days occurred in 0.5%, and renal insufficiency (i.e. estimated glomerular filtration rate <30 ml/min per 1.73 m^2^) was identified as an independent predictor (OR 4.70, 95% CI 1.07–20.61, *p* = 0.040). Among patients undergoing CTO-PCI, 1‑year mortality was 3.7%, and independent predictors included renal insufficiency (OR 5.59, 95% CI 3.25–9.59, *p* < 0.001), left ventricular ejection fraction <30% (OR 3.43, 95% CI 2.00–5.90, *p* < 0.001), previous MI (OR 1.62, 95% CI 1.14–2.31, *p* = 0.007) and age (OR 1.06 per year increment, 95% CI 1.04–1.07, *p* < 0.001). Target-vessel revascularisation <1 year occurred in 11.3%.

**Conclusion:**

CTO-PCI is still infrequently performed in the Netherlands. The most important predictor of mortality after CTO-PCI was renal insufficiency. Identification of patients at risk may help improve the prognosis of CTO patients in the future.

**Electronic supplementary material:**

The online version of this article (10.1007/s12471-020-01521-y) contains supplementary material, which is available to authorized users.

## What’s new?

- The proportion of percutaneous coronary interventions (PCIs) performed for chronic total occlusions (CTOs) is currently still only 6.3%, but the number of procedures has increased in recent years.
- CTO patients are younger, more often male and have more extensive cardiovascular comorbidities than non-CTO patients.
- The presence of renal insufficiency in CTO patients is the most important predictor of mortality and 30-day myocardial infarction, and should be taken into account when referring patients for CTO revascularisation.
- It is proposed that more CTO-PCI-specific parameters should be reported in national PCI databases.

## Introduction

A chronic total coronary occlusion (CTO) is the ultimate expression of coronary artery disease and defined as 100% obstruction of the coronary lumen with complete cessation of antegrade blood flow [1]. CTOs mostly result from progressive atherosclerotic disease, but can also emanate from previous asymptomatic or untreated myocardial infarction (MI) [2]. Accordingly, the incidence of CTOs was rather high in the thrombolysis era, with registries from the 1990s reporting incidences of ~50% in patients undergoing diagnostic coronary angiography [3]. This incidence has decreased over the past two decades, and a CTO is now reported to be present in approximately 20% of patients undergoing coronary angiography and in approximately 15% of patients with acute ST-segment elevation myocardial infarction (STEMI) [4–7]. The presence of a CTO is associated with ischaemic symptoms and poor clinical outcomes, such as impaired left ventricular ejection fraction (LVEF), and increased risk for cardiogenic shock and mortality [6, 8].

Percutaneous coronary intervention (PCI) for a CTO is considered the final frontier for the interventional cardiologist and is performed in only 10% of all CTOs [5]*.* In the Netherlands, the total number of PCI procedures has grown considerably since the early 1990s, while the proportion of CTO procedures has only slightly increased [9]. However, due to novel techniques, improved devices and dedicated high-risk PCI programs and operators, the technical success rates of CTO-PCI are increasing, which allows revascularisation of more complex CTOs [10]. Therefore, the aforementioned numbers may be outdated. Here, we provide the contemporary results of CTO-PCI from a Dutch nationwide registry. We aimed to identify factors that predict the risk of poor outcomes after CTO-PCI to improve patient selection for CTO revascularisation.

## Methods

### Study design

The Netherlands Heart Registration (NHR) is a nationwide registry initiated in 2017, merging three national registries [11]. The NHR registers outcomes of various cardiac interventions, including PCI, from the 30 hospitals in the Netherlands that perform PCIs. Data collection and registration are performed by the participating centres in a secure online environment. In the current study, we included all interventions registered from January 2015 to December 2018 that included a PCI with ≥1 CTO in one of the treated vessels. All interventions with out-of-hospital cardiac arrest, cardiogenic shock or STEMI at admission, as well as all follow-up procedures, were excluded from the analysis. The research protocol of the current study was approved by the NHR Steering Committee. A waiver for informed consent for participation in the NHR was previously obtained from the Ethics Committee.

In this study, we aimed (1) to describe the current rate of CTO-PCI in the Netherlands and (2) to identify risk factors that are predictive of the clinical outcomes 1‑year mortality, target-vessel revascularisation (TVR) <1 year, urgent coronary artery bypass grafting (CABG) <24 h and MI <30 days.

### Definitions

The definitions are in accordance with the NHR Handbook, available via www.nederlandsehartregistratie.nl. A CTO was defined as an atherosclerotic occlusion in one of the treated vessels, older than 3 months and with a thrombolysis in myocardial infarction (TIMI) flow grade of 0 or 1 [1]. Multivessel disease was defined as the presence of a stenosis with >70% luminal narrowing in >2 native major coronary arteries or first-order side branches. Non-ST-segment elevation myocardial infarction (NSTEMI), including unstable angina pectoris, was defined as acute chest pain, in the absence of ST-segment elevation. LVEF was measured at a maximum of 6 months prior to the intervention. When only a descriptive LVEF was provided, this was converted to percentages as follows: good/normal = 55%; moderate = 40%; poor = 25%; severely depressed = 20%. Renal insufficiency was defined as an estimated glomerular filtration rate (eGFR) of <30 ml/min per 1.73 m^2^. TVR was defined as repeat PCI in the index coronary artery during follow-up. The outcome measures were CABG within 24 h after PCI, MI within 30 days after PCI [including NSTEMI and STEMI, excluding periprocedural MI (i.e. type 4 MI, occurring within 48 h after PCI)], TVR within 1 year after PCI and 1‑year mortality after PCI.

### Statistical analysis

Normality of numerical data was determined using visual assessment of histograms. Normally distributed numerical data were described as mean ± standard deviation (SD) and compared using Student’s *t*-test. Non-normally distributed numerical data were described as median with interquartile range (IQR) and compared using the Mann-Whitney U test. Categorical data were described as frequencies with percentages and compared using Fisher’s exact test or the chi-square test. The rate of CTO-PCI was calculated as the total number of CTO-PCIs (including both primary and follow-up procedures, as well as cardiogenic shock, out-of-hospital cardiac arrest and STEMI procedures) relative to the total number of PCIs in the study period, and the change over the study period was tested with the chi-square test. Logistic regression was performed to test the predictive value of baseline characteristics for the outcomes of interest. For variables with >20% of missing data (i.e. LVEF and previous PCI), a dummy category for the missing values was included in the regression model (not shown in the tables). All significant (*p* < 0.05) or relevant predictors from the univariable model were inserted in an enter model for multivariable logistic regression. The impact of the assessed risk factors on outcomes was described as odds ratio (OR) with corresponding 95% confidence interval (CI). For the analysis of 1‑year mortality and 1‑year TVR, all cases with an intervention date in 2018 were excluded, since 1‑year follow-up had not been completed at the time of this study. Survival curves were constructed using the Kaplan-Meier method and compared using the log-rank test. All statistical analyses were performed using SPSS 26 (IBM Corp. Released 2019. IBM SPSS Statistics for Windows, Version 25.0. Armonk, NY, USA).

## Results

### Baseline characteristics

**Fig. 1:**
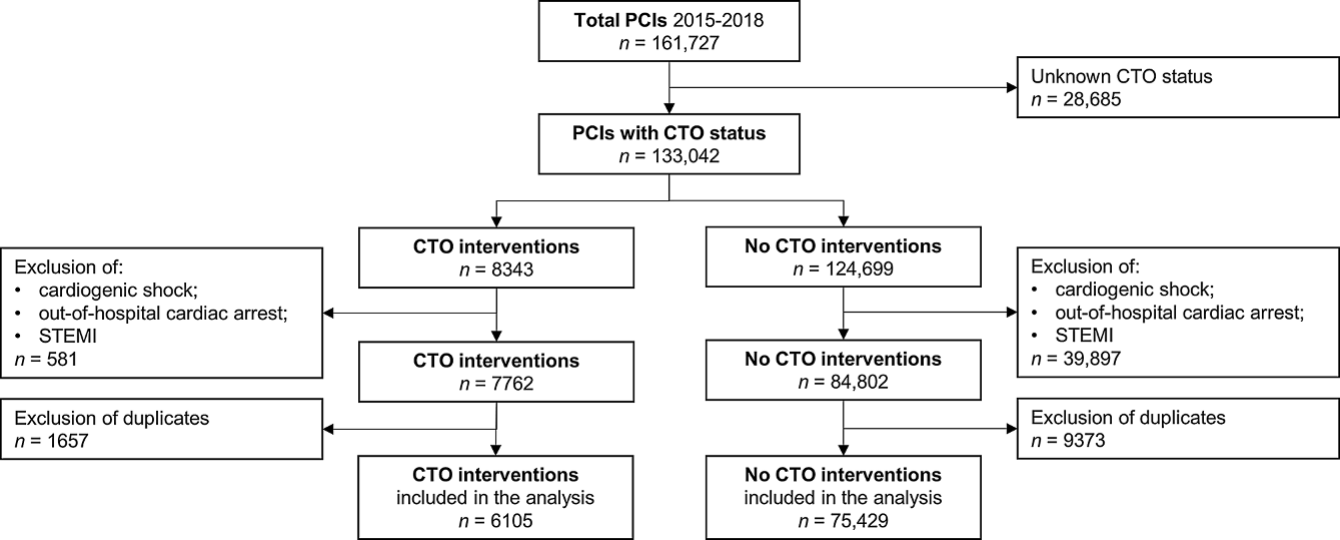
Flowchart of percutaneous coronary intervention (*PCI*) patients registered in the Netherlands Heart Registration between 2015 and 2018. *CTO* chronic total occlusion, *STEMI* ST-segment elevation myocardial infarction

**Fig. 2:**
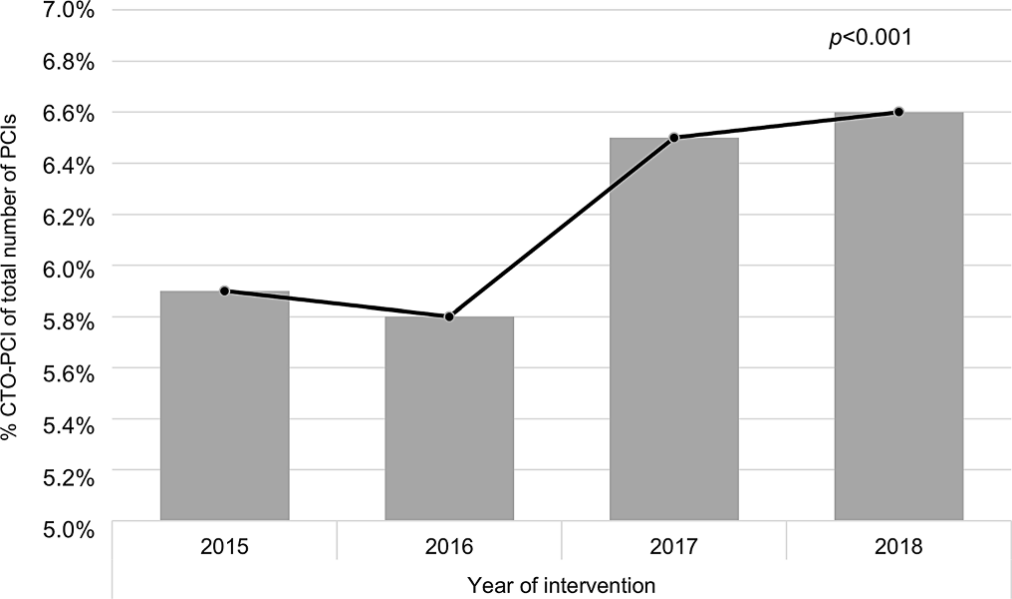
Temporal trend regarding the percentage of percutaneous coronary interventions (*PCIs*) performed for chronic total occlusion (*CTO*)

A total of 161,727 PCI procedures were registered in the NHR, performed between 1 January 2015 and 31 December 2018. In 28,685 procedures the CTO status was not registered. A total of 8,343 of 133,042 procedures (6.3%) included a CTO in one of the treated arteries (Fig. 1). The proportion of CTO-PCIs in the total number of PCIs increased significantly over time from 5.9% in 2015 to 6.6% in 2018 (*p* < 0.001; Fig. 2). After applying the exclusion criteria, 6,105 CTO-PCIs were identified. Baseline characteristics are displayed in Tab. 1. CTO patients were more frequently male (76.8% vs 70.8%, *p* < 0.001), younger (65.7 years vs 67.2 years, *p* < 0.001), more often had diabetes mellitus (25.5% vs 23.4%, *p* < 0.001), reduced LVEF <50% (39.8% vs 33.2%, *p* < 0.001), multivessel disease (55.2% vs 47.6%, *p* < 0.001), previous MI (34.1% vs 26.3%, *p* < 0.001) and previous CABG (14.4% vs 11.1%, *p* < 0.001) than patients without CTO.

**Table 1:** Baseline characteristics of percutaneous coronary intervention (*PCI*) patients with or without chronic total occlusion (*CTO*) registered in the Netherlands Heart Registration

	CTO patients		Non-CTO patients		
		Available Data (*n*)		Available Data (*n)*	*p*-Value
*Age, years*	65.7 ± 10.7	6,105	67.2 ± 11.1	75,429	<0.001
*Male gender*	4,687 (76.8)	6,105	53,406 (70.8)	75,429	<0.001
*Diabetes mellitus*	1,533 (25.5)	6,022	17,321 (23.4)	73,978	<0.001
*Creatinine level, µmol/l (median, IQR)*	86 (IQR 26)	5,841	84 (IQR 27)	70,867	<0.001
*Dialysis*	26 (1.0)	2,692	215 (0.6)	35,173	0.027
*eGFR, ml/min per 1.73* *m*^*2*^		5,848		70,899	0.28
>60	4,459 (76.2)		53,630 (75.6)		
30–59	1,227 (21.0)		15,343 (21.6)		
15–29	105 (1.8)		1,345 (1.9)		
<15 or on dialysis	26 (0.4)		215 (0.3)		
*LVEF, %*		3,697		39,028	<0.001
>50	2,226 (60.2)		26,070 (66.8)		
30–50	1,080 (29.2)		9,968 (25.5)		
<30	391 (10.6)		2,990 (7.7)		
*Indication for PCI*		6,105		75,429	<0.001
Elective	4,841 (79.3)		39,177 (51.9)		
NSTEMI	1,264 (20.7)		36,252 (48.1)		
*Multi-vessel disease*	3,321 (55.2)	6,012	35,639 (47.6)	74,828	<0.001
*Previous MI*	2,048 (34.1)	5,999	19,393 (26.3)	73,717	<0.001
*Previous PCI*	1,126 (31.2)	3,612	13,911 (31.2)	44,637	>0.99
*Previous CABG*	877 (14.4)	6,071	8,268 (11.1)	74,579	<0.001
*Year of intervention*		6,105		75,429	0.25
2015	1,348 (22.1)		15,870 (21.0)		
2016	1,295 (21.2)		16,095 (21.3)		
2017	1,718 (28.1)		21,336 (28.3)		
2018	1,744 (28.6)		22,128 (29.3)		

Data are presented as number of patients (percentages) or mean ± standard deviation, unless otherwise stated. *CABG* coronary artery bypass grafting, *eGFR* estimated glomerular filtration rate, *IQR* interquartile range, *LVEF* left ventricular ejection fraction, *MI* myocardial infarction, *NSTEMI* non-ST-segment elevation myocardial infarction

Procedural characteristics of CTO-PCIs are displayed in Tab. 2. The majority of procedures were performed via the radial artery (67.4%). Very sparse data about the use of double access were available. The most commonly treated artery was the right coronary artery (42.8%), and most interventions included stent placement (83.9%), which could be considered an equivalent for technical success of CTO-PCI in this registry.

**Table 2:** Procedural characteristics of interventions for chronic total occlusions

	Interventions *n* (%)	Available data *n*
*Access route 1*		2,802
Radial artery	1,888 (67.4)	
Femoral artery	902 (32.2)	
Brachial artery	12 (0.4)	
*Double access used*	21 (15.7)	134
*Target artery*		3,050
Left main/left anterior descending	1,111 (36.4)	
Right coronary artery	1,305 (42.8)	
Ramus circumflexus^a^	585 (19.2)	
Arterial/venous bypass graft	49 (1.6)	
*Type of procedure*		2,918
Stent placement	2,448 (83.9)	
Drug-eluting stent	1,725 (70.5)	
Bare-metal stent	5 (0.2)	
Bioresorbable vascular scaffold	1 (0.0)	
Unknown stent type	717 (29.3)	
Balloon dilatation	253 (8.7)	
Aborted procedure	217 (7.4)	

^a^Including the anterolateral/intermediate artery

### Clinical outcomes

**Fig. 3:**
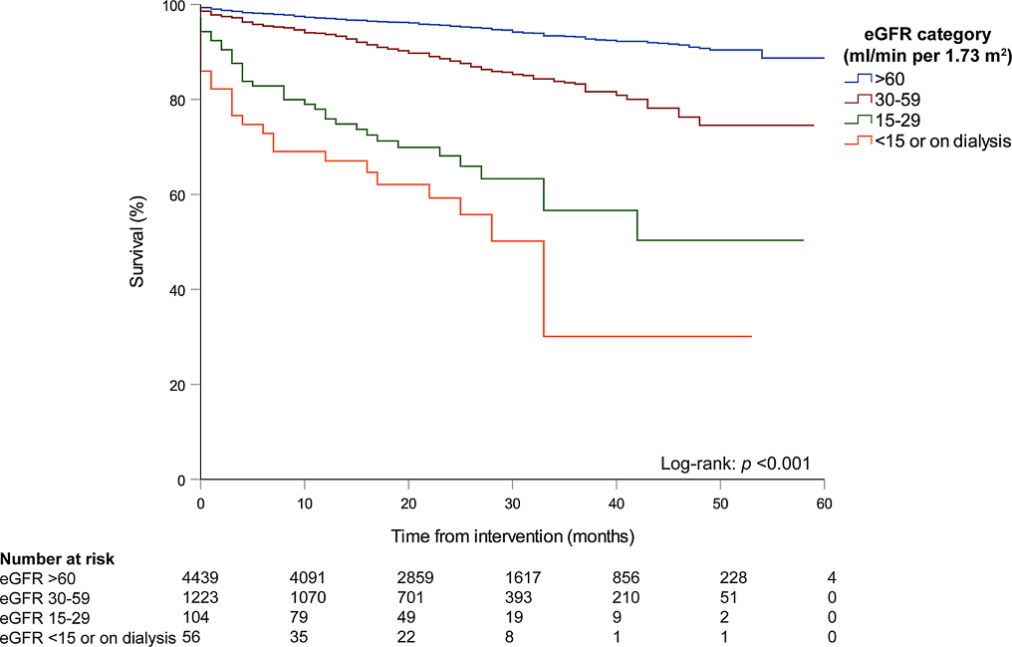
Survival curves, stratified by category of estimated glomerular filtration rate (*eGFR*)

The median duration between intervention and follow-up was 23 months (IQR 16–36). The 30-day mortality was 0.9% (57/6,066) and 1‑year mortality 3.7% (207/5,555). In multivariable logistic regression analysis, the strongest predictor of 1‑year mortality was renal insufficiency (OR 5.59, 95% CI 3.25–9.59, *p* < 0.001; Tab. 3). Patients with eGFR in the lowest category (i.e. eGFR <15 ml/min per 1.73 m^2^ or on dialysis) and the second-lowest category (i.e. eGFR 15–29 ml/min per 1.73 m^2^) had the lowest survival rate (log-rank: *p* < 0.001; Fig. 3). Other independent predictors of 1‑year mortality were LVEF <30% (OR 3.43, 95% CI 2.00–5.90, *p* < 0.001), the presence of previous MI (OR 1.62, 95% CI 1.14–2.31, *p* = 0.007) and age (OR 1.06 per year increment, 95% CI 1.04–1.07, *p* < 0.001). Figure S1 (Electronic Supplementary Material) shows the survival curves stratified for eGFR category for both CTO and non-CTO patients.

**Table 3:** Univariable and multivariable logistic regression model for 1‑year mortality in patients undergoing a percutaneous coronary intervention (*PCI*) for a chronic total occlusion

	Univariable logistic regression				Multivariable logistic regression			
		95% Confidence Interval				95% Confidence Interval		
	OR	Lower Limit	Upper Limit	*p*-Value^a^	OR	Lower Limit	Upper Limit	*p*-Value^a^
*Age, years*	1.06	1.05	1.08	<0.001	1.06	1.04	1.07	<0.001
*Female gender*	1.67	1.18	2.36	0.004	1.36	0.93	1.98	0.11
*Diabetes mellitus*	1.89	1.35	2.65	<0.001	1.34	0.93	1.94	0.12
*LVEF* ^b,c^								
*30–50%*	1.96	1.23	3.11	0.005	1.50	0.91	2.45	0.11
*<30%*	4.06	2.46	6.70	<0.001	3.43	2.00	5.90	<0.001
*Renal insufficiency*	9.64	5.99	15.51	<0.001	5.59	3.25	9.59	<0.001
*Multi-vessel disease*	1.34	0.95	1.87	0.09				
*Previous MI*	1.81	1.31	2.52	<0.001	1.62	1.14	2.31	0.007
*Previous PCI*^c^	1.27	0.79	2.04	0.32				
*Previous CABG*	1.80	1.22	2.67	0.003	1.24	0.80	1.93	0.34
*Presentation with ACS*^d^	1.65	1.16	2.35	0.005	1.04	1.00	1.08	0.07
*Year of intervention*^e^								
2016	0.91	0.62	1.35	0.66				
2017	0.97	0.66	1.44	0.89				

*ACS* acute coronary syndrome, *CABG* coronary artery bypass grafting, *LVEF* left ventricular ejection fraction, *MI* myocardial infarction, *OR* odds ratio

^a^ Significant predictors in the univariable model were entered in the multivariable model. An enter model was used for multivariable logistic regression

^b^ The reference category was LVEF >50%

^c^ Since the amount of missing data was substantial (i.e. >20%) a dummy variable for the missing values was included in the regression model (not shown in the tables)

^d^ Elective procedures versus non-ST-elevation ACS as an indication for PCI

^e^ The reference year was 2015. Interventions in 2018 were excluded from this analysis, as the 1‑year follow-up had not been completed

CABG <24 h occurred in 15 of 5,980 patients (0.3%) and in univariable logistic regression analysis only diabetes mellitus was associated (OR 2.97, 95% CI 1.04–8.49, *p* = 0.042; Electronic Supplementary Material, Table S1). Myocardial infarction <30 days occurred in 19 of 4,214 patients (0.5%). In univariable logistic regression analysis, renal insufficiency was associated (OR 4.70, 95% CI 1.07–20.61, *p* = 0.040) and female gender tended to be associated with MI <30 days (OR 2.45, 95% CI 0.98–6.10, *p* = 0.06; Electronic Supplementary Material, Table S2). TVR <1 year occurred in 489 of 4,336 patients (11.3%). No predictors of TVR could be identified (Electronic Supplementary Material, Table S3).

## Discussion

We present the outcomes of CTO-PCI in the Netherlands from 2015 to 2018. The amount of missing data was substantial for several variables. We found that the percentage of CTO-PCI as a proportion of the total number of PCIs has increased over the study period, although the rate is still only 6.3%. Patients with CTO were younger, more often male and more frequently had diabetes, multivessel disease, reduced LVEF, previous MI and previous CABG, compared to patients without CTO. Patients with renal insufficiency had a 6-fold higher risk for 1‑year mortality and a 5-fold higher risk for 30-day myocardial infarction. Patients with diabetes mellitus had a 3-fold higher risk for urgent CABG <24 h after the procedure.

### Clinical outcomes

Our numbers illustrate that, despite growing success rates and improved techniques, CTO-PCI still remains an infrequently performed procedure. Previously, Hoye et al. reported that from 1992 to 2002 the total number of PCI procedures in a tertiary centre in the Netherlands almost doubled, while the number of CTO-PCIs only slightly increased [9]. The authors reported a CTO-PCI rate of ~10% of all PCI procedures in 2002 at their tertiary care hospital, while we found a rate of only 6.3% in the NHR, which includes both tertiary and secondary care hospitals. This is comparable to the 5.8% reported previously from the nationwide Swedish Coronary Angiography and Angioplasty Registry [7]. Due to its high complexity and success rates of around 80–90%, it is advised that CTO-PCI should be reserved mainly for specialty centres with large volumes and skilled operators [10, 12]. This results in lower nationwide numbers of CTO-PCIs compared to tertiary centres.

The success rates of 80–90%, together with a marked risk for complications, should be weighed against a modest clinical benefit of CTO-PCI over medical therapy alone according to randomised clinical trials [13–16]. These trials demonstrated a significant improvement of symptomatology after CTO-PCI, while mortality rates, the incidence of major adverse cardiac events and left ventricular function did not change when compared to medical therapy alone. We found that renal insufficiency was the strongest predictor of reduced mortality in patients with CTO. It has been demonstrated that chronic kidney disease is a frequent comorbidity among CTO patients, and previous studies reported an incidence of approximately 25%, which is comparable to our data [17, 18]. We demonstrated that CTO patients with renal insufficiency were at a 6-fold higher risk for mortality after PCI compared with patients without renal insufficiency. Particularly patients in the two lowest categories (i.e. eGFR <15 ml/min per 1.73 m^2^ and 15–30 ml/min per 1.73 m^2^) had the worst rate of survival compared to patients with normal or only moderately impaired kidney function. Accordingly, the above-mentioned studies by Moroni et al. and Tajti et al. demonstrated that the presence of chronic kidney disease is associated with an increased risk for both in-hospital and long-term complications after CTO-PCI [17, 18]. In the meta-analysis of Moroni et al. lower procedural and technical success rates of CTO-PCI were also described in this specific patient population [17] while, on the other hand, the exposure of these patients to high contrast doses could lead to an acute or chronic deterioration of their renal function [19]. Hence, in this vulnerable patient population a more restrictive approach with refrainment from PCI could be preferable. It is therefore of great clinical importance to take the presence of renal insufficiency into account when referring patients for CTO-PCI.

### Netherlands Heart Registration

The NHR is a national registry initiated in 2017, in which all cardiovascular interventions performed in the Netherlands are registered. The NHR publishes annual quality reports on the frequencies and outcomes of these interventions for physicians to ensure and improve the quality of cardiovascular care.

A well-maintained nationwide registry has important clinical value in providing real-world data, in addition to controlled clinical trials that adhere to various exclusion criteria. Unfortunately, in the NHR the amount of missing data was substantial for several baseline characteristics as well as some outcome measures, hampering proper data analysis. However, the completeness and accuracy of data input has improved since the inception of the NHR. It should be emphasised that appropriate and complete data input by all physicians and data managers is of great importance to ensure the clinical and qualitative value of a nationwide registry.

### Limitations

Several limitations should be acknowledged. First, since a CTO was defined in the registry as the presence of a CTO in the treated vessel, it cannot be ascertained that the PCI concerned treatment of the CTO. In the most recent audit, performed in 2019 on a total of 2,500 records at 29 sites, a ~ 1% discrepancy was found for the CTO variable between the source documentation and the registration. There, the CTO variable was screened as being the target lesion of the PCI procedure. Therefore, we interpreted this variable accordingly. Second, in the NHR there are currently only a limited number of variables that concern information about the CTO intervention. The recording of more relevant CTO variables could lead to more in-depth analysis of this specific patient population. Suggested CTO variables for inclusion in the NHR are displayed in the Electronic Supplementary Material (Table S4). Third, there is no adjudication of endpoints. However, an audit is randomly performed to ensure data quality and uniformity. Lastly, no control group of CTO patients not undergoing PCI was included; therefore, comparisons between treatment strategies could not be made.

## Conclusion

In this nationwide registry, we found that the number of CTO-PCIs is increasing, although the procedure is still infrequently performed. CTO patients were younger, more often male and more frequently had reduced LVEF and concurrent cardiovascular disease compared with patients without CTO. Renal insufficiency was identified as the most important predictor of 1‑year mortality and 30-day myocardial infarction, and diabetes mellitus was associated with urgent CABG <24 h after the CTO procedure. Identification of patients at risk for poor clinical outcomes could optimise the referral of appropriate patients for CTO revascularisation.

## Electronic Supplementary Material

Supplemental Tables and Figure.
