# Supplementary material for: Incidence and outcomes of chronic total occlusion percutaneous coronary intervention in the Netherlands: data from a nationwide registry
Source: Neth Heart J. 2020 Dec 2;29(1):4–13. doi: 10.1007/s12471-020-01521-y (PMC7782624; doi:10.1007/s12471-020-01521-y)
Supplement: Supplementary file 1 — Supplemental Tables and Figure. [file 12471_2020_1521_MOESM1_ESM.docx]

**ELECTRONIC SUPPLEMENTARY MATERIAL**

**Table S1.** Univariable logistic regression model for coronary artery bypass grafting <24 hours in CTO-PCI patients

**Table S2.** Univariable logistic regression model for 30-day myocardial infarction in CTO-PCI patients

**Table S3.** Univariable logistic regression model for 1-year target-vessel revascularisation in CTO-PCI patients

**Table S4.** Suggested new CTO variables to include in the NHR

**Fig. S1.** Survival curves of CTO and no-CTO patients, stratified by eGFR category

**Table S1** Univariable logistic regression model for coronary artery bypass grafting <24 hours in CTO-PCI patients

|  | Univariable logistic regression | | | |
| --- | --- | --- | --- | --- |
|  |  | **95% confidence interval** | |  |
|  | **OR** | **Lower limit** | **Upper limit** | **p-value** |
| Age, years | 0.97 | 0.93 | 1.02 | 0.19 |
| Female gender | 2.21 | 0.79 | 6.22 | 0.13 |
| Diabetes mellitus | 2.97 | 1.04 | 8.49 | 0.042 |
| LVEF ^a,b^ | 1.02 | 0.96 | 1.08 | 0.58 |
| 30-50% | 0.51 | 0.11 | 2.43 | 0.40 |
| <30% | 0.71 | 0.09 | 5.70 | 0.75 |
| Renal insufficiency | 0.00 | 0.00 | - | >0.99 |
| Multi-vessel disease | 2.26 | 0.72 | 7.12 | 0.16 |
| Previous MI | 2.22 | 0.81 | 6.14 | 0.12 |
| Previous PCI ^b^ | 0.00 | 0.00 | - | 0.99 |
| Previous CABG | 0.44 | 0.06 | 3.37 | 0.43 |
| Presentation with ACS ^c^ | 0.95 | 0.27 | 3.35 | 0.93 |
| Year of intervention ^d^ |  |  |  |  |
| 2016 | 1.30 | 0.26 | 6.43 | 0.75 |
| 2017 | 2.25 | 0.54 | 9.44 | 0.27 |
| 2018 | 1.46 | 0.33 | 6.52 | 0.62 |

ACS = acute coronary syndrome; CABG = coronary artery bypass grafting; LVEF = left ventricular ejection fraction; MI = myocardial infarction; OR = odds ratio; PCI = percutaneous coronary intervention. ^a^ The reference category was LVEF >50%. ^b^ Since the amount of missing data was substantial (i.e. >20%) a dummy variable for the missing values has been included in the regression model (not shown in Table). ^c^ Elective procedures versus non-ST-elevation ACS as indication for PCI. ^d^ The reference year was 2015.

**Table S2** Univariable logistic regression model for 30-day myocardial infarction in CTO-PCI patients

|  | Univariable logistic regression | | | |
| --- | --- | --- | --- | --- |
|  |  | **95% confidence interval** | |  |
|  | **OR** | **Lower limit** | **Upper limit** | **p-value** |
| Age, years | 1.02 | 0.97 | 1.06 | 0.46 |
| Female gender | 2.45 | 0.98 | 6.10 | 0.06 |
| Diabetes mellitus | 2.21 | 0.89 | 5.51 | 0.09 |
| LVEF ^a,b^ | 0.99 | 0.95 | 1.02 | 0.41 |
| 30-50% | 1.08 | 0.32 | 3.58 | 0.91 |
| <30% | 1.45 | 0.31 | 6.86 | 0.64 |
| Renal insufficiency | 4.70 | 1.07 | 20.61 | 0.040 |
| Multi-vessel disease | 1.76 | 0.67 | 4.65 | 0.25 |
| Previous MI | 1.45 | 0.58 | 3.61 | 0.43 |
| Previous PCI ^b^ | 1.31 | 0.38 | 3.04 | 0.86 |
| Previous CABG | 1.08 | 0.31 | 3.71 | 1.08 |
| Presentation with ACS ^c^ | 1.54 | 0.56 | 4.30 | 0.41 |
| Year of intervention ^d^ |  |  |  |  |
| 2016 | 0.73 | 0.18 | 2.91 | 0.65 |
| 2017 | 1.01 | 0.28 | 3.59 | 0.99 |
| 2018 | 1.07 | 0.34 | 3.31 | 0.91 |

ACS = acute coronary syndrome; CABG = coronary artery bypass grafting; LVEF = left ventricular ejection fraction; MI = myocardial infarction; OR = odds ratio; PCI = percutaneous coronary intervention. ^a^ The reference category was LVEF >50%. ^b^ Since the amount of missing data was substantial (i.e. >20%) a dummy variable for the missing values has been included in the regression model (not shown in Table). ^c^ Elective procedures versus non-ST-elevation ACS as indication for PCI. ^d^ The reference year was 2015.

**Table S3** Univariable logistic regression model for 1-year target-vessel revascularisation in CTO-PCI patients

|  | Univariable logistic regression | | | |
| --- | --- | --- | --- | --- |
|  |  | **95% confidence interval** | |  |
|  | **OR** | **Lower limit** | **Upper limit** | **p-value** |
| Age, years | 1.00 | 0.99 | 1.01 | 0.31 |
| Female gender | 0.90 | 0.70 | 1.16 | 0.41 |
| Diabetes mellitus | 1.12 | 0.88 | 1.41 | 0.35 |
| LVEF ^a,b^ |  |  |  |  |
| 30-50% | 1.21 | 0.92 | 1.60 | 0.18 |
| <30% | 0.86 | 0.56 | 1.32 | 0.48 |
| Renal insufficiency | 1.29 | 0.71 | 2.35 | 0.40 |
| Multi-vessel disease | 1.21 | 0.97 | 1.49 | 0.09 |
| Previous MI | 1.00 | 0.80 | 1.25 | 0.98 |
| Previous PCI ^b^ | 0.98 | 0.67 | 1.43 | 0.91 |
| Previous CABG | 1.14 | 0.86 | 1.52 | 0.37 |
| Presentation with ACS ^c^ | 0.87 | 0.67 | 1.13 | 0.30 |
| Year of intervention ^d^ |  |  |  |  |
| 2016 | 1.08 | 0.84 | 1.39 | 0.54 |
| 2017 | 1.14 | 0.89 | 1.47 | 0.30 |

ACS = acute coronary syndrome; CABG = coronary artery bypass grafting; LVEF = left ventricular ejection fraction; MI = myocardial infarction; OR = odds ratio; PCI = percutaneous coronary intervention. ^a^ The reference category was LVEF >50%. ^b^ Since the amount of missing data was substantial (i.e. >20%) a dummy variable for the missing values has been included in the regression model (not shown in Table). ^c^ Elective procedures versus non-ST-elevation ACS as indication for PCI. ^d^ The reference year was 2015. Interventions in 2018 were excluded from the analysis, since those did not yet complete the 1-year follow-up.

**Table S4** Suggested new CTO variables to include in the NHR

| **Variable name** | **Description** |
| --- | --- |
| CTO presence, *yes/no* | Is a CTO present in one of the major coronary arteries or 1^st^ order side branches? |
| CTO location, *number of coronary segment* | In which coronary artery segment is the CTO located? |
| CTO intervention, *yes/no* | Does the current intervention concern a PCI of the CTO? |
| J-CTO score: blunt stump, *yes/no* | Is the entry blunt or tapered (i.e. tapered tip or dimple)? |
| J-CTO score: calcification, *yes/no* | Is any evidence of calcification present (regardless of severity)? |
| J-CTO score: bending >45 degrees, *yes/no* | Is a bending of >45 degrees detected in the CTO segment? Any tortuosity separated from the CTO segment is excluded. |
| J-CTO score: occlusion length ≥20 millimeters, *yes/no* | Is the “true” length of the occlusion ≥20 mm? |
| J-CTO score: re-try (2^nd^ or more attempt), *yes/no* | Is this a re-try lesion? I.e. previously attempted but failed. |
| J-CTO: total score^1^ | (Auto-calculated) 0 = easy; 1 = intermediate; 2 = difficult; 3 or more = very difficult |
| CTO access route, *single access/double access* | Was a single access route or double access route used? |
| CTO technique 1 used, *AWE, ADR, RWE, RDR* | Which technique was used? |
| CTO technique 2 used, *AWE, ADR, RWE, RDR* | Which technique was used? *(Optional if more than 1 technique was used)* |
| CTO residual stenosis, *%* | What was the residual stenosis of the target CTO? |
| CTO post-procedural TIMI flow | What was the TIMI flow post-CTO procedure? |
| CTO intervention successful (according to the operator), *yes/no* | Was the CTO intervention technically successful, according to the operator? |
| CTO intervention successful, *yes/no* | (Auto-calculated)  Technical success, defined as TIMI flow grade of ≥2 and less than 30% residual stenosis of the target lesion. |
| CTO modes of failure, *inability to cross the proximal cap; inability to cross the lesion with a balloon after successful wire crossing; inability to dilate the balloon; re-entry failure; loss of distal visualization; abortion due to major complication; other, specify…* | If unsuccessful CTO procedure, what was the main reason for failure? |

ADR = antegrade dissection and re-entry; AWE = antegrade wire escalation; CTO = chronic total occlusion; J-CTO = Japan CTO; PCI = percutaneous coronary intervention; RDR = retrograde dissection and re-entry; RWE = retrograde wire escalation; TIMI = Thrombolysis in Myocardial Infarction.

^1^ Morino Y, *et al*: **Predicting successful guidewire crossing through chronic total occlusion of native coronary lesions within 30 minutes: the J-CTO (Multicenter CTO Registry in Japan) score as a difficulty grading and time assessment tool**. *JACC Cardiovascular interventions* 2011, **4**(2):213-221.

**Fig. S1** Survival curves of CTO and no-CTO patients, stratified by eGFR category


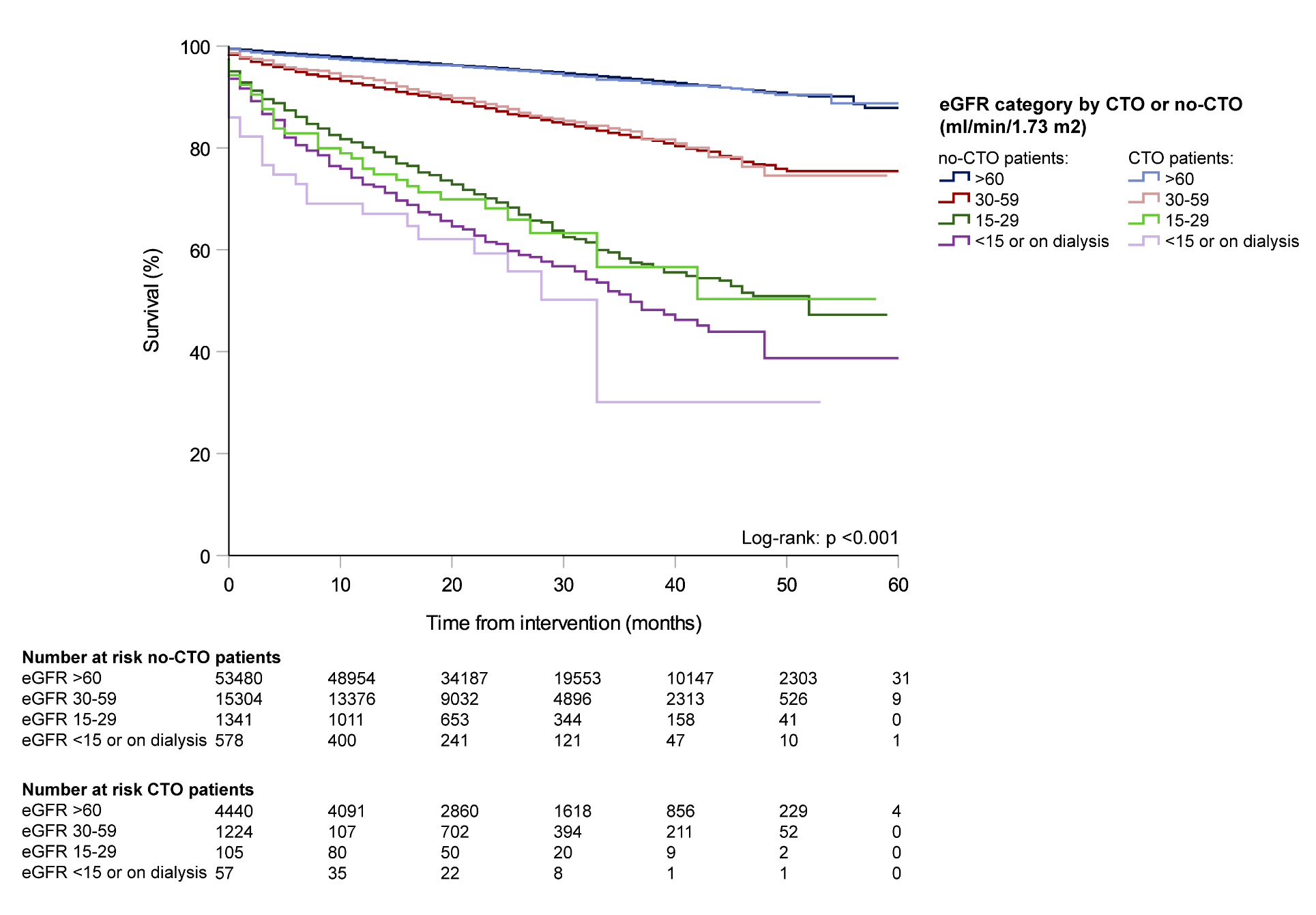

CTO = chronic total occlusion; eGFR = estimated glomerular filtration rate.
